# Critical illness in prisons: a multi-methods analysis of reported healthcare safety incidents in England

**DOI:** 10.3399/BJGP.2025.0239

**Published:** 2026-01-13

**Authors:** Isobel J McFadzean, Lauren Donovan, Thomas Hewson, Jake Hard, Jenny Shaw, Adrian Edwards, Andrew Carson-Stevens

**Affiliations:** 1 Division of Population Medicine, School of Medicine, Cardiff University, Cardiff, UK; 2 Northwest School of Psychiatry, Manchester, UK; 3 South West Prisons, Oxleas NHS Foundation Trust, London, UK; 4 Independent Advisory Panel on Deaths in Custody, England, UK; 5 Division of Psychology and Mental Health, University of Manchester, Manchester, UK; 6 PRIME Centre Wales, Cardiff, UK

**Keywords:** critical illness, emergencies, general practice, health care, patient safety, prisons

## Abstract

**Background:**

Prisoners have disproportionately poorer health and complex needs compared with the general population. Prisons should provide care that is equivalent to community care to achieve equitable health outcomes, which includes managing physical deterioration.

**Aim:**

To characterise reported patient safety incidents involving critically unwell prisoners and identify opportunities to improve prison healthcare systems.

**Design and setting:**

Secondary multi-methods analysis of incident reports submitted from English prisons to the National Reporting and Learning System (NRLS) between 2018 and 2019.

**Method:**

The patient safety incidents had been characterised in previous research focusing on patient safety in prisons, describing incident types, contributory factors, and outcomes. Purposive sampling of these coded data was carried out using search terms to identify healthcare-associated harm, or near misses, related to critical illness (ill health with risk of death if urgent care is not provided). Included reports were sequentially analysed by descriptive and framework analysis.

**Results:**

Of 4112 reports submitted to the NRLS within 12 months, 983 (23.9%) were identified by the search terms and screened, and 94 (9.6%) met the inclusion criteria for analysis, containing 189 safety incidents. The most common patient outcomes within the reports included delayed assessment or treatments (*n* = 46 reports, 48.9%), avoidable hospital admissions (*n* = 15, 16.0%), and patient deterioration (*n* = 13, 13.8%). Key issues identified were insufficient provision of emergency equipment, failure to recognise severity of symptoms and act appropriately on symptoms, and ineffective communication between prisons and ambulance services. Moderate and severe harm outcomes were reported in a quarter of reports (*n* = 26, 27.7%).

**Conclusion:**

System-wide interventions are needed to improve the safety of care delivered to critically ill prisoners, including improved continuity of care, enhanced emergency response training, reviews of emergency protocols surrounding clinical assessments, recognition of critical illness, escalation plans, and communication with wider teams.

## How this fits in

Using a multi-methods descriptive and framework analysis, this study provides new insights into the complexity of care delivery in prisons. Results resonate with and strengthen the recommendations from recent investigations into prison health care by further developing an understanding of the complex intersecting factors contributing to safety incidents and quality issues in care delivery. The fundamental importance of good quality and adequately resourced primary care delivery in prisons is highlighted. Also identified are system-wide interventions that are needed to improve care delivery. This will likely interest policymakers and scrutiny bodies, commissioners, and teams working in prisons to inform developments in strategic health needs assessments, workforce profiling, and training requirements for healthcare and prison teams.

## Introduction

There are concerns regarding the safety of health care received by prisoners in the UK, owing to the environment, risk of injury, self-harm and death, and suboptimal service delivery.^1–3^ Prison populations are ageing, with primary care teams including general practitioners (GPs), nurses, pharmacists, and allied healthcare professionals such as dentists, optometrists, and podiatrists delivering the majority of care to prisoners,^4^ managing both acute and long-term conditions with access to prisoner medical records,^5^ while balancing security measures and healthcare standards.^6^ Health care is regularly delivered on site, or referrals sent to external healthcare teams as needed. All prisons carry out health screening of new prisoners to establish their healthcare needs, and some have dedicated healthcare wings with inpatient beds.^7,8^ Furthermore, staff shortages and prison lockdowns can have an impact on healthcare access, and appointments are regularly missed because of insufficient officer escort numbers for transfers to hospital appointments.^6,9,10^ There is growing trepidation that prisoners may deteriorate clinically, and conditions worsen, before receiving timely care,^11^ with high rates of substance use complicating care delivery despite security measures in place to prevent access to substances.^12^ Additionally, prisoners can be motivated to mismanage their health conditions, to gain priority when healthcare resources are limited, and/or to transfer to lower security settings or healthcare wings, for example, prisoners with type 1 diabetes mellitus refusing insulin to invoke diabetic ketoacidosis and hospital admission.^13^

Healthcare professionals and allied workers within prisons are encouraged to record events in which patients were, or could have been, harmed while receiving health care, in the form of patient safety incidents.^14–17^ Analysis of these reports supports a greater understanding of healthcare quality and safety, aiming to comprehend any contributory factors of safety events, to support improvements.^6^ Researchers have also begun to investigate rates of healthcare-associated harm within prisons,^18^ and the Health Services Safety Investigations Body (HSSIB) in England has assessed continuity of care^9^ and emergency care provision within secure environments.^19^ The HSSIB found that prisoners frequently cannot access necessary health care, and inefficient transfer of information between healthcare teams affects treatment and/or delays ongoing referrals.^9^ This accords with the authors’ previous research analysing incident reports in English prisons,^6,20^ alongside issues identified by the independent health think tank, The Nuffield Trust,^21^ from their focus on prisoner health and promoting evidence-based medicine to improve the quality of health care within secure environments.^11,22^

The National Confidential Enquiry into Patient Outcome and Death (NCEPOD) found that prisoners die, on average, approximately 20 years earlier than the wider population.^23^ While ‘natural causes’ remain the main documented cause of death, conclusions following the Independent Advisory Panel on Deaths in Custody report found that many deaths were preventable and could not be solely attributed to an ageing population. They concluded preventable deaths occurred because of poor healthcare management such as an inability to recognise deteriorating patients.^24^ The NCEPOD also highlighted deficits in anticipating emergencies, escalating care to emergency services, competently delivering first aid, and accessing emergency support and equipment.^23^

The principle of ‘equivalence’ within secure environments, as recommended by the Royal College of General Practitioners in the UK,^25^ and the United Nations standard minimum rules for the treatment of prisoners,^26^ advocates that prisoners are entitled to healthcare standards equivalent to community care to support equitable health outcomes. This includes managing acute and long-term conditions and adhering to nationally accepted clinical guidelines where appropriate. Despite this ambition, in comparison with primary care within community settings, prisoners currently receive poorer quality care in England.^27^

An understanding of the safety of health care for critically unwell prisoners would identify key areas for improvement, with prisoner health being a primary care and public health concern, however, there is a paucity of research into this.^19^ The aim of this study was therefore to characterise reported patient safety incidents involving critically unwell prisoners and identify opportunities to improve prison healthcare systems. The key objectives to achieve this aim were to:

- identify and characterise reported patient safety incidents involving critically unwell prisoners by their clinical conditions; and

- identify contributory factors to harm arising from reported safety incidents involving critically unwell prisoners.

## Method

### Setting

Data were collected from patient safety incidents reported to the National Reporting and Learning System (NRLS), a national repository of safety incident reporting,^16^ from all English prisons, including those with remand (holding individuals charged but not convicted) and training functions (providing education, vocational skills, and rehabilitation programmes),^28^ over a 12-month period from 1 April 2018 to 31 March 2019. This period was chosen to inform a wider study , investigating the epidemiology of avoidable healthcare-associated harm in prisons,^18^ and to allow an insight into critical illness management within secure environments, before any additional constraints, for example, the COVID-19 pandemic.^29^

### Sample

A secondary analysis of a previous retrospective multi-method analysis was completed, exploring patient safety incidents within prisons. This dataset captured incidents reported by secure environments using location search terms ‘prison/remand’, requesting all reports submitted to the NRLS over the 12-month period.

After reviewing all reports, key terms and synonyms that captured critical illness events were listed, including critical conditions, hospital transfer, and paramedic involvement such as ‘epilepsy’, ‘ambulance’, and ‘999’, respectively. These terms were applied to all 4112 reports (see Supplementary Table S1) and academic GP clinicians within the study team (the first and second authors) read the reports to make a judgement about whether they were about critical illness.

### Inclusion criteria

Inclusion criteria were:

- reports that met the definition of a patient safety incident, defined by the NHS as, *‘any unintended or unexpected incidents which could have, or did, lead to harm for one or more patients while receiving healthcare’*;^16^
- incidents that originated within a prison, and contained sufficient information to determine what happened (incident type) and perceived reasons why the incident and resulting harm or near miss might have occurred (contributory factors); and
- reports that involved a ‘critical illness’ based on an existing definition that the authors contextualised for the prison context and nature of the data:^30^ *‘a state of ill health with vital organ dysfunction, a high risk of imminent death if care is not provided’.*

### Coding

All incident reports had previously undergone systematic coding^6^ recorded by academic GPs (the first author and Kate Davies [see Acknowledgements]) using the Patient Safety (PISA) classification system^31^ that is comprised of several coding frameworks, and used to classify incident type(s) (what happened), contributory factor(s) (why), incident outcome(s) (patient impact), and harm severity. Coding occurred within the PISA database, a bespoke Structured Query Language (SQL) database.^32^ The PISA classification system is aligned with the ontology for patient safety, as described in the World Health Organization’s (WHO) International Classification for Patient Safety,^33^ and up to four incident types, contributory factors, and/or outcomes were coded for each incident within the PISA database.

Incident reports often describe multiple incidents or complex care journeys; therefore all coded data from the primary study were reviewed, and amended, to focus on incidents involving critical illness only, with the illness and/or conditions classified by the first and the second author, according to the 11th edition of the International Classification of Diseases (ICD).^34^ Only what was explicitly stated within the free-text was coded, using the recursive model of incident analysis.^35^ To assess interrater reliability, a Cohen kappa coefficient was calculated to determine agreement between the first and second author for 20% of their coded reports.

An exploratory descriptive analysis of the coded data supported development of quantitative summaries, and cross-tabulation of variables in Microsoft Excel (version 16.0) enabled identification of the most frequently occurring relationships between critical illness condition, incident types, contributory factors, and outcomes.

### Framework analysis

Framework analysis supported a systematic process for managing and analysing the qualitative data, while still allowing flexibility to incorporate both *a priori* themes, and emergent insights from the data. First, all included incident reports with contributory factors were uploaded to the software NVivo (version 12) and re-read to support data familiarisation. New themes were identified and indexed by the second author.

PISA contributory factor codes and emergent themes were then summarised and mapped to the six domains of the Systems Engineering Initiative for Patient Safety (SEIPS) by academic GPs (the first author and Tom Purchase). The six domains of SEIPS are: ‘tools and technology’ (accessibility, functionality, and maintenance), ‘internal environment’ (physical environment characteristics), ‘tasks’ (actions within larger work processes), ‘organisation’ (time, space, resources, and activity), ‘person’ (individual characteristics), and ‘external environment’ (societal, economic, regulatory, and policy factors externally).^36^ This process enabled identification of the range and intensity of specific factors or themes within and across the SEIPS domains, supporting a structured approach to understanding how components of the healthcare system interact to influence safety.^37,38^

## Results

**Figure 1.**
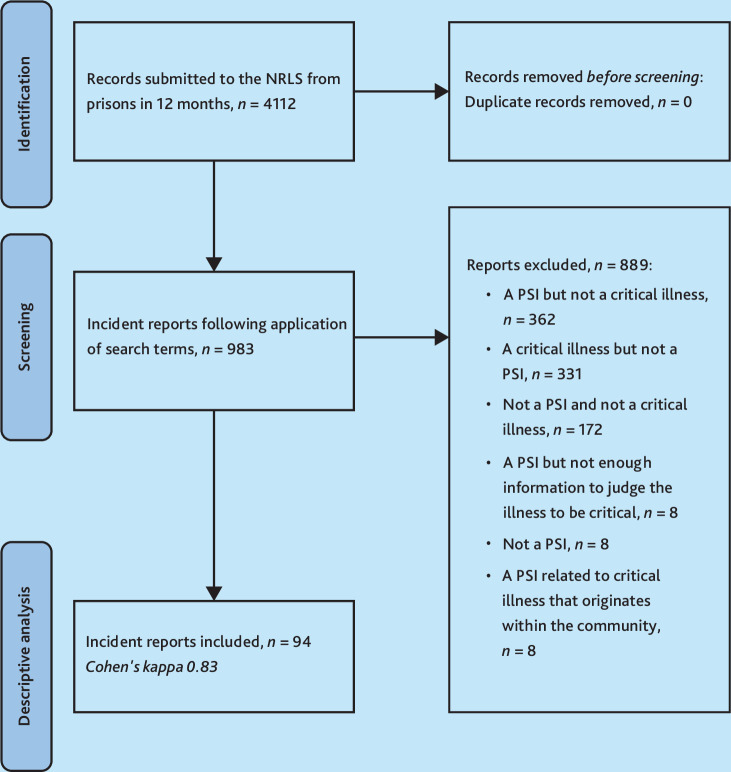
Flow diagram of sample formation. NRLS = National Reporting and Learning System. PSI = patient safety incident.

Of the original 4112 reports submitted to NRLS from English prisons, searches of the dataset yielded 983 (23.9%) reports, and nearly 10% (*n* = 94) of these met the inclusion criteria for detailed analysis (see Figure 1). A third of reports (*n* = 362, 36.8%) were excluded from focused analysis because they were patient safety incident reports but did not relate to critical illness, and another third (*n* = 331, 33.7%) were excluded because they were not detailed enough to meet the definition of a patient safety incident, for example, reports in which there was no mention of a healthcare incident, but instead a brief statement that a patient had self-harmed and required hospital admission, while receiving appropriate management.

A kappa of 0.83 for report inclusion was calculated, indicating near perfect agreement between the first and second author.

### Clinical conditions

Patient safety incidents occurred while prisoners experienced a range of critical illnesses and conditions, and the most frequently reported involved sequelae relating to substance use (*n* = 11/94, 11.7%), epilepsy/seizures, and cardiac arrests (both *n* = 10, 10.6%, respectively). By organ system, cardiovascular (*n* = 23, 24.5%), neurological (*n* = 16, 17.0%), and mental health conditions (*n* = 14, 14.9%) were most commonly described (see Supplementary Table S2 for all coded illnesses organised by body system, and the associated incident types).

### Incident type

Most reports contained multiple incident types (*n* = 70, 74.5%), with 189 incidents identified in total (see Supplementary Table S3 for the top incident types and associated contributory factors). Nearly one-sixth of incidents related to delays accessing healthcare professionals (*n* = 23, 12.2%). Such delays involved accessing emergency care/secondary care physicians (*n* = 10/23, 43.5%), a quarter (*n* = 6, 26.1%) related to accessing paramedics, and nearly a quarter related to nursing staff (*n* = 5, 21.7%). Next were problems with insufficient treatment, care, or monitoring (*n* = 15/189, 7.9%). Of these, nearly half involved medication administration (*n* = 4, 26.7%) or the wrong medication being administered (*n* = 3, 20.0%), specifically involving antibiotics such as flucloxacillin, anti-epileptic medication, and diazepam for the management of seizures. This was followed by observations not being carried out as requested by healthcare professionals (*n* = 3, 20.0%) and overdoses not escalated for further care or monitoring (*n* = 2, 13.3%).

Other commonly reported incidents involved emergency transport issues (*n* = 10/189, 5.3%) and insufficient assessment of the patient (*n* = 10, 5.3%). From reports where insufficient assessment was identified, most (*n* = 7/10, 70.0%) related to insufficient physical assessment of the patient, whereas the remaining reports (*n* = 3, 30.0%) involved insufficient mental state assessments. Failure to act appropriately on symptoms (*n* = 10/189, 5.3%) was also commonly reported, including responses to seizures (*n* = 2/10, 20.0%), post-ligature (*n* = 2, 20.0%), and symptoms suggestive of a stroke (*n* = 1, 10.0%) (see Supplementary Table S3).

All coded incidents can be found within Supplementary Table S4 and example reports are given in Box 1.

Box 1. **Example reports**

- *‘Patient collapsed in the dormitory of the in-patient unit. They were known to have epilepsy and had a recent, serious head injury. There were no officers on the healthcare unit with a cell key to enable the nurse to access the patient and assess them … ’*
- *‘This patient had been reviewed having been reported to have symptoms of a stroke, with right sided facial palsy, headache and tinnitus ... Despite an Emergency Department referral being done, patient was not taken immediately last night as requested and was sent by car this morning. I have been informed of this by email today, and we will discuss this at our next quality meeting.’*
- *‘There were numerous agency nurses working at HMP* [redacted] *and patient* [redacted] *had a “fall” and sustained significant head injuries. No clinical or neurological observations were taken by staff, and the patient began to have several seizures and was admitted to hospital. We have seeked* [sic] *guidance from* [Human Resources] *and have been advised that the staff member no longer works at this prison … ’*
- *‘The information that a prisoner had taken a large overdose was not shared with the healthcare team until over four hours later, this resulted in a very late admission to the Emergency Department … ’*
- *‘This patient was known to use substances and has epilepsy, with a high risk of seizures. They collapsed and required treatment for sepsis, however there was a delay calling for an ambulance, and when the paramedics arrived, they took over 30 minutes to take them to hospital, despite the nurses knowing that they should have insisted immediate treatment and transfer.’*

### Contributory factors

From the 94 reports, the majority (*n* = 87, 92.6%) contained at least one contributory factor, defined as issues that did not directly cause but contributed to the occurrence of an incident,^31^ or ‘why’ an incident occurred, with 152 contributory factors identified overall. The most frequent factor related to prison environment/prison context (*n* = 32/152, 21.1%), including access constraints and security lockdowns. Working conditions, including short-staffed healthcare teams (*n* = 23, 15.1%), several emergencies occurring at once (*n* = 23, 15.1%), and continuity of care (*n* = 17, 11.2%), specifically with critical medication prescribing for conditions such as epilepsy and diabetes, were common factors (see Supplementary Table S5 for all contributory factors).

### Outcomes

There were 127 outcomes coded in total, defined as *‘the impact upon a patient which is wholly or partially attributable to an incident’* ^39^ and nearly half of the incidents (*n* = 46/127, 36.2%) resulted in delays in management, assessment, or treatment. Next were avoidable hospital admissions (*n* = 15, 11.8%) or unclear outcomes (*n* = 15, 11.8%), and then general deterioration or progression of the condition (*n* = 13, 10.2%) (see Supplementary Table S6 for all outcomes).

### Harm severity

Actual harm could not be determined by the study team in most reports, categorised as ‘unclear’ harm (*n* = 54/94, 57.4%), but where harm was described, half resulted in moderate harm (*n* = 21/40, 52.5%). Of 94 reports, five incidents (5.3%) resulted in severe harm, with no deaths directly associated with the incidents (see Supplementary Table S7 for all harm severities).

### Framework analysis

#### Thematic development

**Figure 2.**
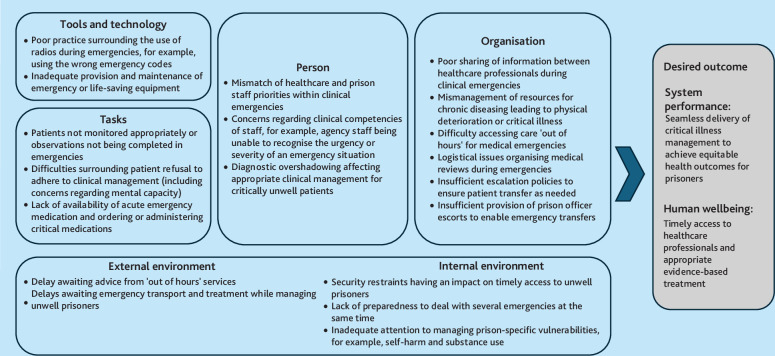
Themes mapped to domains of the Systems Engineering Initiative for Patient Safety.

The mapping of contributory factors to SEIPS (see Supplementary Figure S1) was used as a lens to aid the interpretation and development of themes and sub-themes, which were also mapped to appropriate SEIPS domains (see Figure 2). Examples of reports linked with sub-themes can be found within Supplementary Box S1.

Contributory factors and sub-themes aligned with SEIPS informed the identification of six overarching themes (described in Supplementary Box S2):

- prisoners unable to access healthcare professionals when required;
- difficulties arising from the vulnerabilities of the prison population;
- insufficient and/or inadequate recognition of, and response to, critical illness with escalation when required;
- ineffective teamworking within prison settings;
- poor provision and maintenance of life-saving equipment and medication; and
- issues with pathways and transfer of health information across the healthcare system.

These themes cut across all SEIPS domains, demonstrating the complexity of patient safety incidents involving critical illness in prisons.

## Discussion

### Summary

This study identified incidents occurring to patients with a range of critical illnesses and conditions, notably complications arising from substance use, epilepsy, self-harm, and injuries. Prisoners experienced delays accessing healthcare professionals, problems with transport logistics, and poor management, assessment, and/or treatment. Conflicting perspectives and priorities of prison staff and healthcare teams were evident contributors to unsafe care, particularly disagreements about condition severity. Continuity of care was also problematic, with issues transferring information, service provision, and management of long-term conditions. Non-adherence to protocols and guidelines surrounding emergencies, poor communication with ambulance services regarding details of events, and inadequate availability of equipment to manage emergencies were identified. The layout of prisons and access to unwell prisoners also contributed, coupled with overwhelmed healthcare staff, and insufficient access to care during evenings and weekends.

### Strengths and limitations

To the authors’ knowledge, this is the first national characterisation of reported incidents relating to the management of critical illness in prisons. Adopting an internationally advocated ‘systems-based approach’ to understand patient safety incidents^40–45^ enabled an appreciation of the likely contributory factors to, and related complexity of, addressing patient safety incidents in prisons. Contributory factors and key themes mapped to SEIPS highlight vulnerabilities across multiple and frequently occurring factors across system domains and has enriched understanding about how future improvements can be made. The limitations of incident reporting are well known, namely selection, reporting, and hindsight biases,^29^ alongside variable quality data, for example, the severity of harm within the majority of prison reports was unclear, reflecting perhaps an emphasis of quantity over quality, and incident reporters not always having all of the facts to hand when writing these incident reports.^46^ Similarly, owing to the anonymity and sensitivity of the datasets and the volume of reports included for analysis it was not possible to comment on themes occurring within specific prisons nor cross-cutting themes across such prisons or prison categories, with future work needed to address this. However, the present study demonstrated the hypothesis-generating potential of such data to inform safety improvement agendas, and other methods such as case-note review will be able to corroborate such observations, determine their frequency, and build a more complete picture of care delivery in this context.

### Comparison with existing literature

Although there is a paucity of information on the management of critical illnesses within primary care, a systematic review and meta-analysis of incident reports in intensive care units globally exemplified that critically unwell patients are at a higher risk of experiencing patient safety incidents.^37^ Efforts to identify and mitigate system issues during the delivery of critical care in hospital environments include creating a cultural shift with a focus on healthcare safety, and ensuring psychological safety within organisations.^38^ However, these changes may be difficult to implement in prisons, given their distinctiveness and balance of health care and security, with focus instead needed to support decision making, diagnostic reasoning, and critical thinking to reduce these events, perhaps through regular simulation training.^47^

The challenges of delivering safe prison health care are well recognised,^48,49^ with a high physical and mental health condition prevalence among prisoners^50^ and a concerning number of drug-related deaths^51–54^ or suicides.^55,56^ The current study has demonstrated that the safety consequences arising from these vulnerabilities are complex, and the factors cross-cut SEIPS domains, in particular ‘internal environment’, ‘tasks’, and ‘person factors’, demonstrating how several areas would need to be addressed to mitigate future harm.

Additionally, the challenges of managing long-term conditions in prisons has been reported.^9,23^ This study has deepened understanding about ineffective team working and concerns regarding continuity of care, particularly during transfers between prisons or to/from hospital settings, with delayed outpatient appointments and resulting healthcare deterioration. A transferrable digital patient record information system^57^ could mitigate risks associated with information transfer and poor continuity of care in prisons.

Another study used the SEIPS framework to explore medication-related incidents in intensive care environments, demonstrating how stress, communication problems, and knowledge deficits collectively influence safety incidents.^58^ However, the systemic factors are likely to be different and thus solutions to address them are unlikely to be transferable. The prison context is complicated by the systemic constraints arising from security considerations, and it is unclear how effective solutions proposed in other contexts, such as supported decision making, diagnostic reasoning, and critical thinking can reduce such events.^59^ The present research has also exposed the interaction of common factors across a wider range of patient safety incident types suggesting that interventions to mitigate harm will need to be complex to address a myriad of sociotechnical factors.

There were concerns surrounding communication between prison officers, healthcare teams, and ambulance services. Other research exploring delivery of mental health services in prison showed a ‘disconnect’ between prison officers and healthcare teams because of prison officers prioritising security protocols, which can conflict with healthcare worker aims.^60–62^ While the authors explored the multifactorial nature of incidents, the importance of staff factors was evident, highlighting the link between team conflicts and patient safety incidents, with issues related to working conditions, locum/agency staff, and team disagreement. This is perhaps indicative of the culture within secure environments, with research indicating high levels of staff burnout and poor attitudes towards, and understanding of, prisoner health, including self-harm and mental health.^63^

### Implications for research and practice

Following a review of all contributory factors and emergent themes guided by a human-factors approach, the study team, made up of patient safety experts, a GP with prison experience, and forensic psychiatrists, considered how to address these issues, developing recommendations, as seen within Box 2. Implementing these recommendations may be challenging across the custodial estate but they are essential to improve patient safety and ensure equitable health outcomes for people who reside in prisons.

Box 2. **Recommendations**

- Appropriate levels of staffing, for both prison healthcare and prison officer teams must be ensured, to reflect workload and particularly to cope with the risk-prone contexts that have been observed, such as the high turnover of prisoner patients, and single and mass incident responses
- Immediate life support training contextualised for the prison environment through simulation training is required for both prison officers and healthcare staff
- Implementing the provision of reliable and clear routes of escalation for healthcare teams where there are specific patient safety concerns
- Establishing clear protocols for access to healthcare services within and outside of normal working hours
- Greater attention to healthcare appointment systems to minimise missed appointments
- Improved digital record information system infrastructure to minimise incidents arising from failures in record transfer
- Prioritisation of emergency equipment availability, with clear expectations of roles and responsibilities for ensuring equipment maintenance and access

In conclusion, priorities for improving the safety of care delivered to critically unwell prisoners to implement care equivalence, include measures to strengthen team cohesion and effective working within the prison context and with outside agencies (such as ambulance services) during emergencies, as well as reliable access and maintenance of emergency equipment. The multifactorial and complex range of systemic factors within and external to prisons that underpin critical illness-related safety incidents, highlights that interventions to improve patient safety involving critical illness in prisons require codevelopment between different professional groups and agencies.

## Data

A data sharing agreement was created between Cardiff University and NHS England (reference: DSA 5131). Incident reports used for the study are available by application to NHS England only. The IDs of the reports used for the study can be provided on reasonable request.
